# Effect of glyphosate and P on the growth and nutrition of *Coffea arabica* cultivars and on weed control

**DOI:** 10.1038/s41598-021-87541-z

**Published:** 2021-04-14

**Authors:** Yanna Karoline Santos da Costa, Nagilla Moraes Ribeiro, Guilherme Cesar Pereira de Moura, Artur Rodrigues Oliveira, Silvano Bianco, Ricardo Alcántara-de la Cruz, Leonardo Bianco de Carvalho

**Affiliations:** 1 School of Agricultural and Veterinarian Sciences, São Paulo State University (UNESP), Jaboticabal, São Paulo 14884-900 Brazil; 2 Chemistry Department, Federal University of São Carlos, São Carlos, SP 13565-905 Brazil

**Keywords:** Plant sciences, Environmental sciences

## Abstract

The effect of the phosphorus (P) and glyphosate interactions on the growth and nutrition of Arabica coffee cultivars (*Coffea arabica*), as well as on the control of *Ipomoea grandifolia* and *Urochloa decumbens*, was evaluated. Catuaí-Amarelo/IAC-62 and Catuaí-Vermelho/IAC-144 cultivars did not show glyphosate poisoning, regardless of the soil P content. However, glyphosate reduced the growth of Catuaí-Vermelho/IAC-144. In addition, the soil P content influenced the height, leaf area and dry matter of Catuaí-Amarelo/IAC-62, and the absorption of P and Ca in both cultivars. On the other hand, glyphosate efficiently controlled *U. decumbens* but not *I. grandifolia*. Glyphosate effectiveness on *I. grandifolia* decreased as the soil P content increased. In addition, the soil P content and the glyphosate influenced the P content in *I. grandifolia* and *U. decumbens* plants. The soil P content influenced the growth and absorption of other nutrients by coffee plants as well as glyphosate effectiveness on weed control.

## Introduction

Coffee is one of the most popular drinks in the world, being its grains one of the most valuable commodities globally^1^. In Brazil, coffee cultivation occupied 3.4 million ha in 2020, generating jobs in rural properties, foreign exchange and tax collection^1, 2^. Arabica coffee (*Coffea arabica*) is most valued for providing a better-quality drink and lower caffeine content, accounting for 58% of the world supply, while Conilon coffee (*C. canephora*) shares 42% of the coffee market^3^.

Coffee production in tropical regions takes place in highly weathered soils with low availability of phosphates (Pi), making it necessary to apply high amount of phosphorous (P) fertilizers to maintain agricultural production^4^. P of these fertilizers can be precipitated by iron and aluminum or adsorbed and/or immobilized on constituents of the soil, mainly clays, reducing the availability of Pi to be absorbed by plants, which can significantly limit coffee production^4, 5^. In addition, Arabica coffee plants do not tolerate weed interference both at the beginning of its development and during its vegetative, flowering and fruiting phases, compromising productivity^6, 7^. Among the most common weeds that occur in coffee plantations are *Ipomoea grandifolia*, which is widely distributed in Brazilian agricultural fields^7^, and *Urochloa decumbens* because it is used as a cover crop in the interlines of the crop^8^.

Herbicide availability for weed management in coffee is reduced, forcing the use of non-selective herbicides, which have to be applied in a directed jet avoiding contact with the coffee plants^9^. Due to its great availability, low cost and application flexibility, glyphosate is the most widely used herbicide in this crop^10, 11^. However, in adverse environmental conditions that favor drift, glyphosate can reach coffee trees directly by accidental application, or indirectly by spraying causing damage to young plantations^9^, being one of the main obstacles arising from the application of this herbicide in coffee.

Glyphosate has a Pi group in its molecule, therefore, numerous factors can influence the sorption and desorption of glyphosate in the soil, including P ion content, as there is a close relationship between glyphosate and Pi sorption capacity by soils, i.e., the sorption mechanisms of Pi and glyphosate are similar, competing with each other for the same soil retention site^12–15^. On the other hand, plants absorb P through the cell membrane which is translated by Pi transporters, while glyphosate can enter plants by passive diffusion or via endoplasmic transport system, also using Pi transporters from the cell membrane^14, 16^. In some cases, the transport of the saturable component of glyphosate can be competitively inhibited in the presence of Pi^15^; therefore, Pi concentration can affect the absorption of glyphosate in plants^11–13, 17, 18^.

Studies focused on understanding the interaction between glyphosate and P and their effects on plants are scarce; therefore, there is a need to conduct studies to verify whether the Pi fertilization, depending on the amount of fertilizer P, influences the selectivity of coffee to glyphosate, inducing different responses in relation to seedling growth and nutrient absorption, as well as weed control (i.e., *I. grandifolia* and *U. decumbens*), as there is evidence that Pi increased glyphosate absorption by the root in *Hydrocharis dubia* and *Salix miyabeana*, preventing oxidative stress caused by glyphosate^14, 18^. In contrast, *Eucalyptus grandis* plants subject to P deficiency absorbed more ^14^C-glyphosate^12^. These divergences may be associated with the physical–chemical characteristics of the spray solution, since the electrical conductivity, pH, surface tension and viscosity can interfere in the droplet spectrum and influence the interaction of the herbicide with the target surface^19, 20^.

The objective of this work was to evaluate the physical–chemical characteristics of the herbicide spray solutions and the responses regarding the growth and nutrition of young plants of Arabica coffee cultivars, *I. grandifolia* and *U. decumbens* submitted to different concentrations of glyphosate and P.

## Results

### Herbicide solution characterization

The pH and electrical conductivity of the herbicide solutions and the surface tension of the drop varied according to the glyphosate concentration (g acid equivalent (ae) ha^−1^). The higher the glyphosate concentration, the higher the acidity and electrical conductivity, while the surface tension of the drops decreased. However, the viscosity of the herbicide solutions was higher at a dose of 90 g ha^−1^ of glyphosate (Supplementary Fig. S1).

### Response of arabica coffee cultivars to glyphosate and soil P content

The coffee cultivars Catuaí-Amarelo/IAC-62 and Catuaí-Vermelho/IAC-144 did not show signs of intoxication by the herbicide, regardless of the glyphosate subdose (0, 90 or 180 g ae ha^−1^), soil P content available (7, 15, 40 and 172 mg Pi dm^−3^) and time of evaluation (7, 14, 21, 28, 35, 42, 63, 77, 84 and 90 days after treatment—DAA).

### Effect of P and glyphosate on coffee growth

**Figure 1:**
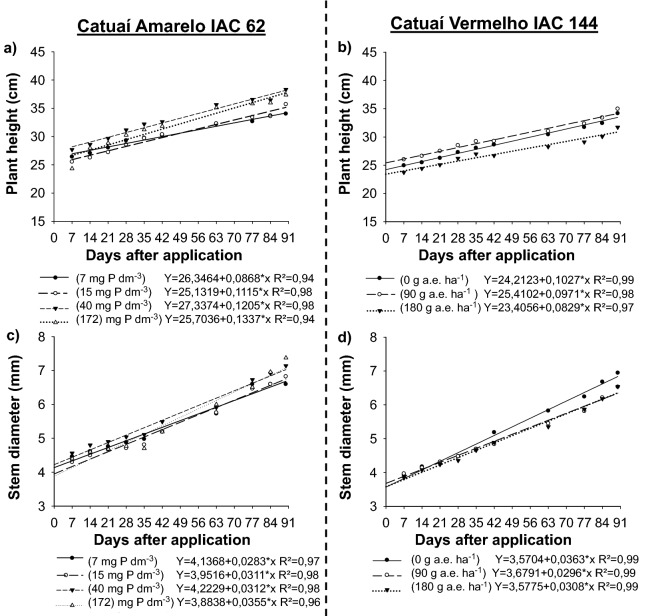
Plant height (cm) (**a**,**b**) and stem diameter (mm) (**c**,**d**) of the arabica coffee cultivars Catuaí-Amarelo/IAC-62 and Catuaí-Vermelho/IAC-144 cultivated in soils with different content of P (mg dm^−3^) and subjected to subdoses of glyphosate in relation to the days after the application of glyphosate. Plots were drawn using SigmaPlot ver. 10.0 (Systat Software, Inc., San Jose, USA, www.systatsoftware.com).

The P content in the soil affected the grow of Catuaí-Amarelo/IAC-62 up to 90 DAA, while for Catuaí-Vermelho/IAC-144, this parameter was affected only by glyphosate. The highest plant height and stem diameter of Catuaí-Amarelo/IAC-62 was observed in soils with 40 and 172 mg Pi dm^−3^, respectively, while the lowest height (24 cm) and diameter (4.3 mm) was found in soils with 7 mg Pi dm^−3^ (Fig. 1a,c). The height of Catuaí-Vermelho/IAC-144 plants differed between glyphosate doses (Fig. 1b,d), recording the highest height (34.9 cm) in plants treated with 90 g ha^−1^ of glyphosate. However, the largest stem diameter (6.94 mm) was recorded in untreated plants (control).

The number of leaves of the cultivar Catuaí-Amarelo/IAC-62 at 35, 77 and 84 DAA varied depending on the soil P content, whereas for Catuaí-Vermelho/IAC-144, this parameter was not affected by the treatments (Supplementary Fig. S2). However, the defoliation observed in the arabica coffee plants during the study was due to the incidence of *Cercospora coffeicola*, which was controlled with application of the fungicide Opera (epoxiconazole + pyraclostrobin, systemic, 1.5 L pc ha^−1^, Basf, Brazil).

**Figure 2:**
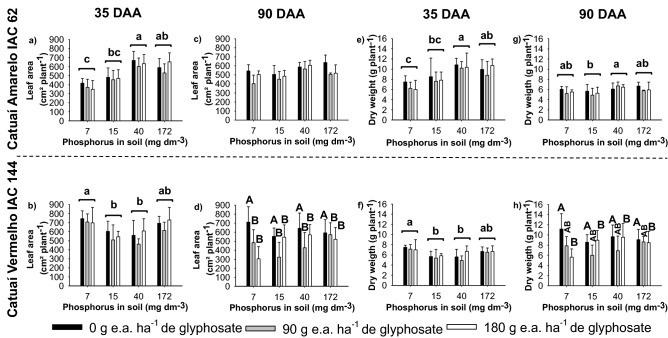
Leaf area and dry matter of Arabica coffee cultivars Catuaí-Amarelo/IAC-62 (**a**,**c**,**e**,**g**) and Catuaí-Vermelho/IAC-144 (**b**,**d**,**f**,**h**), cultivated in soils with different levels of P and subjected to subdoses of glyphosate at 35 and 90 days after application (DAA). For each variable and evaluation time, same lower-case does not differ between the soil P contents, and same upper-case do not differ between the subdoses of glyphosate by the Tukey test at 5% probability. Vertical bars indicate the standard deviation (*n* = 4). Plots were drawn using SigmaPlot ver. 10.0 (Systat Software, Inc., San Jose, USA, www.systatsoftware.com).

The leaf area and dry matter of the aerial part of Catuaí-Amarelo/IAC-62 deferred depending on the soil P content (Fig. 2a,e,g). Plants grown in soils with 40 and 172 mg Pi dm^−3^ had an average of 635 and 591 cm^2^ plant^−1^ of leaf area, respectively, at 35 DAA and of 585 and 554 cm^2^ plant^−1^ at 90 DAA (Fig. 2c). On the other hand, the Catuaí-Vermelho/IAC-144 plants had different responses between the periods of evaluation (Fig. 2b,d,f,h). At 35 DAA, the levels of P were significant for leaf area (p = 0.01) (Fig. 2b) and dry matter (p = 0.007) (Fig. 2f), and at 90 DAA, glyphosate was significant for these variables (p = 0.0099 and p = 0.0206, respectively) (Fig. 2d,h). At 35 DAA, the highest leaf area (717 cm^2^ plant^−1^) (Fig. 2b) and dry matter of the aerial part (7.5 g plant^−1^) (Fig. 2f) was observed in plants grown in soils with 7 mg Pi dm^−3^ but not treated with glyphosate (control). In addition, glyphosate did not affect the plants of Catuaí-Vermelho/IAC-144, as there was no intoxication. At 90 DAA, plants without glyphosate application had an average of 625 cm^2^ plant^−1^ of leaf area and 9.6 g plant^−1^ of dry matter (Fig. 2d,h).

### Nutrient content

At 35 DAA, the interaction between glyphosate doses and soil P content did not influence the accumulation of N, S and P levels in the aerial parts of Catuaí-Amarelo/IAC-62 (Supplementary Fig. S3a,b,d) and the S, Ca, P, K and Mg in Catuaí-Vermelho/IAC-144 (Supplementary Fig. S3h–l). For both arabica coffee cultivars, glyphosate did not affect the P content in the shoots. However, the P content in the plant was higher in those grown in soils with 40 and 172 mg Pi dm^−3^ (Supplementary Fig. S3d,j).

For plants of Catuaí-Amarelo/IAC-62, there was an interaction between the soil P content and glyphosate doses for the levels of K, Ca and Mg (Supplementary Fig. S3c,e,f). The K content varied according to the glyphosate subdose applied, and plants grown in soils with 15 mg Pi dm^−3^ treated with 90 g ha^−1^ of glyphosate, had lower (16 g kg^−1^ plant^−1^) content of K, differing from the other treatments (Supplementary Fig. S3e). The Ca content was lower (9.5 g kg^−1^ plant^−1^) in plants grown in soil with 7 mg Pi dm^−3^, regardless of regardless of glyphosate sub-dose (Supplementary Fig. S3c). Plants grown in 172 mg Pi dm^−3^ and without herbicide application, presented low Mg content in the shoots (Supplementary Fig. S3f).

For plants of Catuaí-Vermelho/IAC-144, lower N levels in the plant were found in coffee grown in soil with 15 and 40 mg Pi dm^−3^ treated with 90 g ha^−1^ glyphosate at 35 DAA (Supplementary Fig. S3g). Lower Ca content was found in non-treated and treated plants with 180 g ha^−1^ glyphosate, cultivated in soils with 7 and 15 mg Pi dm^−3^ (Supplementary Fig. S3i). The P content in Catuaí-Vermelho/IAC-144 plants was higher in soils with 40 and 172 mg Pi dm^−3^ (Supplementary Fig. S3j).

**Figure 3:**
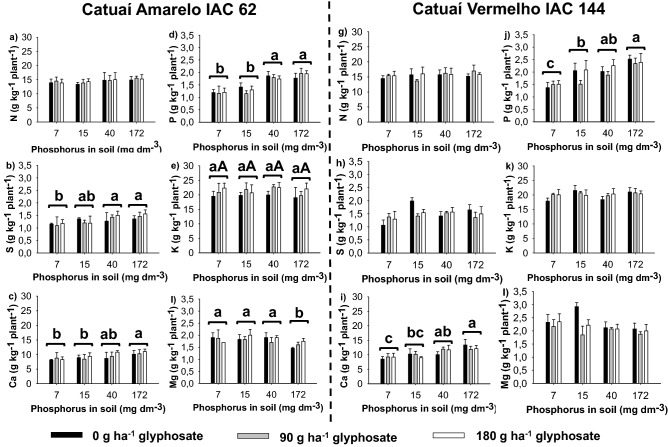
Average nutrient content of the shoot (g kg^−1^ plant^−1^) of the arabica coffee cultivars Catuaí-Amarelo/IAC-62 and Catuaí-Vermelho/IAC-144, cultivated in soils with different levels of P and subjected to subdoses of glyphosate at 90 days after application. (**a**,**g**) Nitrogen; (**b**,**h**) sulfur; (**c**,**i**) calcium; (**d**,**j**) phosphorus; (**e**,**k**) potassium; and (**f**,**l**) magnesium. For each nutrient, same lower-case do not differ between the soil P contents, and same upper-cases do not differ between the glyphosate subdoses by the Tukey test at 5% probability. ± Vertical bars indicate the standard deviation (*n* = 4). Plots were drawn using SigmaPlot ver. 10.0 (Systat Software, Inc., San Jose, USA, www.systatsoftware.com).

At 90 DAA, the different soil P content influenced the levels of S (p = 0.0014), Ca (p = 0.0026), P (p < 0.0001) and Mg (p = 0.0058) in plants of Catuaí-Amarelo/IAC-62 (Fig. 3b–l), and of Ca and P in Catuaí-Vermelho/IAC-144 plants (Fig. 3i,j). There was no interaction between the soil P content and glyphosate for the two cultivars. The cultivars had similar responses when grown in soils with 40 and 172 mg Pi dm^−3^, with higher levels of P and Ca (Fig. 3).

### Response of weeds to glyphosate and soil P content

**Figure 4:**
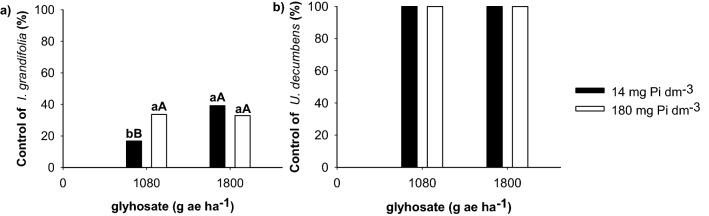
Control percentage of *Ipomoea grandifolia* (**a**) and *Urochloa decumbens* (**b**) plants cultivated in soils with 14 and 180 mg Pi dm^−3^ and submitted to doses of 0, 1080 and 1800 g ha^−1^ of glyphosate at 28 days after application (DAA). Means followed by the same lower-case do not differ between the soil P contents, and means followed by the same upper-case do not differ between the doses of glyphosate by the Tukey test at 5% probability. Plots were drawn using SigmaPlot ver. 10.0 (Systat Software, Inc., San Jose, USA, www.systatsoftware.com).

Glyphosate, regardless of the dose and soil P content did not control *I. grandifolia*, but it was possible to observe that in soils with 180 mg Pi dm^−3^, plants were more susceptible to glyphosate at 1080 g ha^−1^ at 28 DAA. *Urochloa decumbens* plants were controlled by the herbicide, regardless of the dose and soil P content, with plant death (100%) at 21 DAA (Fig. 4).

**Figure 5:**
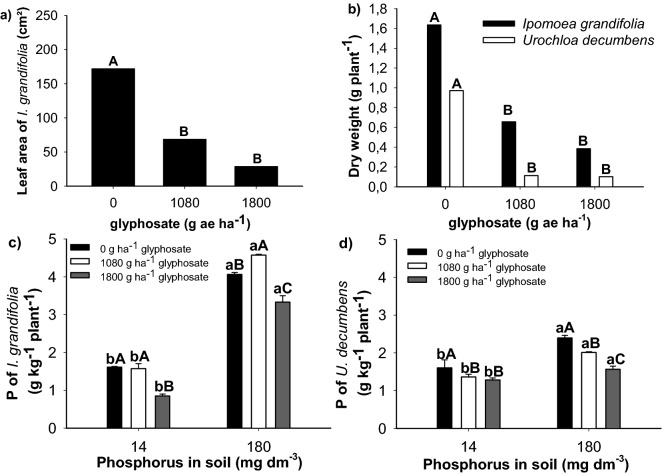
Leaf area of *Ipomoea grandifolia* (**a**) and dry matter of the aerial part of *I. grandifolia* and *Urochloa decumbens* (**b**) in relation to glyphosate doses. P content in the aerial part of *I. grandifolia* (**c**) and *U. decumbens* (**d**) in relation to the soil P content (14 and 180 mg Pi dm^−3^) and doses of glyphosate (0, 1080 and 1800 g ea ha^−1^). For each variable and weed, same lowercase does not differ between the soil P content, and same uppercase do not differ between doses of glyphosate by the Tukey test at 5% probability. ± Vertical bars indicate the standard deviation (n = 7). Plots were drawn using SigmaPlot ver. 10.0 (Systat Software, Inc., San Jose, USA, www.systatsoftware.com).

The leaf area of *I. grandifolia* was not affected by the soil P content (p = 0.4761), but if for glyphosate (p = 0.002), i.e., as the herbicide dose increased the leaf area of *I. grandifolia* decreased (Fig. 5a). The leaf area of *U. decumbens* was carried out only in the control plants (untreated), as plants treated with glyphosate died, where the leaf area increased with the increase of P in the soil (Supplementary Fig. S4). The dry matter of the aerial part of *I. grandifolia* (p = 0.02) and *U. decumbens* (p = 0.002) decreased as the glyphosate doses increased, regardless of the soil P content (Fig. 5b).

The P content in *I. grandifolia* and *U. decumbens* plants was influenced by the amount of soil P content and the glyphosate doses (Fig. 5c,d). Although the P content in the plants was higher when grown in soil with 180 mg Pi dm^−3^, *I. grandifolia* plants accumulated more P when they were treated with 1080 g ha^−1^ of glyphosate, regardless of the soil P content (Fig. 5c), while *U. decumbens* accumulated more P in non-herbicide treated plants (Fig. 5d).

## Discussion

The pH reduction of the spray solutions as the glyphosate doses increased possibly was due to that the Pi and carboxylic groups of the herbicide have a greater acid character than ammonium^21^. Therefore, higher concentrations of the glyphosate decrease the pH improving the herbicide effectiveness, corroborating that the pH of the spray solution interferes in the activity of glyphosate^22^, reducing the surface tension of the droplets with a higher concentration of glyphosate improves the spreading of the spray solution on the leaves, but differences in the epidermis of the cultivars can affect the spraying^20^. The increase in electrical conductivity as glyphosate concentration in g ae ha^−1^ increased may be related to the increase in free ions in the spray solution influencing the herbicide effectiveness on plants^21, 23^. Viscosity, which is correlated to the size of drops at the moment of spraying^23^, increased as the glyphosate dose increased; therefore, the higher viscosity the greater the size of drops and possibly less risk of drift^19^.

The coffee cultivars Catuaí-Amarelo/IAC-62 and Catuaí-Vermelho/IAC-144 showed no signs of intoxication by glyphosate. This may be associated to the relative low glyphosate doses compared to those applied to control weeds. Studies carried out with lower soil P content (2.3 mg Pi dm^−3^) and application of glyphosate subdoses in different cultivars of arabica coffee (Acaiá/MG-6851, Catucaí Amarelo/2SL, Topázio/MG-1190, Oeiras MG-6851) reported signs of intoxication in plants by the herbicide and attributed that there may be differential tolerance between coffee cultivars to the herbicide^24–26^. Therefore, probably the P content in the soil may have influenced the reduced intoxication.

Cultivars Catuaí-Amarelo/IAC-62 and Catuaí-Vermelho/IAC-144 had different growth responses, since the leaf area and dry matter accumulation was smaller in Catuaí-Vermelho/IAC-144 at 90 DAA as the glyphosate doses increased. This difference was possibly due to the differential ability between these coffee cultivars to metabolize or degrade the herbicide into less toxic or non-toxic compounds^27^. However, some herbicides may not cause signs of intoxication, but they can compromise plant growth and development for the rest of the crop cycle^26^. As observed for the plant height, diameter, number of leaves, leaf area and accumulation of dry matter of the aerial part in Catuaí-Vermelho/IAC-144 cultivated in soil with 26 mg Pi dm^−3^ and submitted to 180 or 360 g ha^−1^ of glyphosate^2^. In addition, it was observed that cultivars of Acaiá, Catucaí-Amarelo and Oeiras submitted to 460 g ha^−1^ of glyphosate had a reduction in height, leaf area and dry matter increase of these cultivars^25, 26^, reinforcing that there is a differential response among coffee cultivars.

The number of leaves in both coffee cultivar was no affect by glyphosate. This may be related to the fact that the subdoses of the herbicide were not enough to cause injury to the plants. Similar results were observed in the cultivars Catuaí/IAC-144, Acaiá/IAC 479-19 and Catucaí-Amarelo/2SL, that were also submitted to glyphosate subdoses (57.2, 115.2, 230.4 g ha^−1^)^26, 28^.

The content of nutrients in the cultivars Catuaí-Amarelo/IAC-62 and Catuaí-Vermelho/IAC-144 was influenced by soil P content, but not by the subdoses of glyphosate. In addition, this response differed between coffee cultivars. These coffee cultivars have already been classified as less efficient and responsive to the supply of Pi in the soil compared to the cultivars Acaiá/IAC-474-19, E16-Shoa/IAC-2027 and E22-Sidamo/IAC-2032, when cultivated in soils with low (8 mg Pi dm^−3^) and high (120 mg Pi dm^−3^) P content^4^. The cultivars Catuaí-Amarelo/IAC-62 and Catuaí-Vermelho/IAC-144 accumulated more P and Ca when cultivated in soils with higher P content, but Catuaí-Amarelo/IAC-62 plants were more efficient in accumulating S and Mg in soils with higher P content. On the other hand, the application of glyphosate reduced the levels of N, P and K in leaves of Catucaí, Oeiras, Topázio^29^ and Catuaí-Vermelho/IAC-99^30^, increased the Ca content of these cultivars^24, 30^, and no affected the accumulation of N, P, K, Ca, Mg and S contents in Catuaí-Vermelho/IAC-144^31^. The nutrient accumulation of these cultivars was influenced by glyphosate (360 g ae ha^−1^) to which they were submitted. Summarizing, the coffee plants may preset differential response to the soil P content and glyphosate concentration, depending on the cultivar, as observed in the Catuaí-Amarelo/IAC-62 and Catuaí-Vermelho/IAC-144 cultivars assessed in this study.

The poor control of *I. grandifolia*, regardless of the soil P content, was due to the natural tolerance that this species presents to glyphosate, which is responsible for the reduced absorption and translocation of the herbicide^32^. Plants grown in soils with a higher P content, the efficiency of glyphosate was lower and the P content in the plant increased. This suggests that the soil P content reduces the effectiveness of glyphosate in controlling *I. grandifolia*. The applied dose, age and size of weeds, spray volume and water quality, can also influence the effectiveness of the herbicide^33^. Glyphosate doses of 460 and 920 g ha^−1^ controlled *I. grandifolia* by 78 and 99%, respectively, with reduced dry matter at 35 DAA^34^. In another study, the growth *of I. grandifolia*, grown on a commercial substrate, was reduced by 50% with 615 g ha^−1^ of glyphosate at 21 DAA^35^. These results showed that the tolerance of *I. grandifolia* to glyphosate may increase or decrease due to the soil P content.

*Urochloa decumbens* was controlled (100%) with 1080 g ha^−1^ glyphosate in both soil P concentrations (14 and 180 mg Pi dm^−3^), which reduced the P content in plants compared to *I. grandifolia.* This reduction may be related to the shorter time for P absorption by *U. decumbens* plants, since symptoms of chlorosis and necrosis were observed from 7 DAA. The increase of PO_4_^3−^ fertilization in soils treated with glyphosate leads to an increase in the availability of the herbicide for absorption by the roots, contributing to the efficiency of glyphosate^11, 36^, due to the main Pi sorption sites are surfaces of iron and aluminum oxides, misordered aluminum silicates and edges of layer silicates, while the sorption of glyphosate by permanently charged layer silicates appears to be limited^11, 37^. Therefore, glyphosate may have greater absorption and faster translocation in leaves and roots of plants grown in the absence of Pi^12^. This difference in the glyphosate absorption between the culture media with and without Pi is attributed to the phosphate transporters present, since the expression of the high affinity transporters can increase in the absence of Pi^12^. We emphasize that the loss of effectiveness of glyphosate applied via foliar can be reduced when plants are grown in soils with a high Pi content, contributing to the selection of glyphosate resistant weed biotypes^12^. However, further studies are needed to better understand the relationship of soil P content with glyphosate and its effects on coffee cultivars and weed control, as well as the competition between P and glyphosate in plants, since there may be a differential response between weed species and cultivars.

We concluded that: (i) arabica coffee cultivars Catuaí-Amarelo/IAC-62 and Catuaí-Vermelho/IAC-144 showed tolerance to glyphosate subdoses and differentiated responses in growth to the soil P content; (ii) the soil P content influenced the content of P, S, Ca and Mg of the Catuaí-Amarelo/IAC-62 cultivar and P and Ca in Catuaí-Vermelho/IAC-144, and the glyphosate subdoses affected the growth in Catuaí-Vermelho/IAC-144; (iii) the application of 1080 g ha^−1^ glyphosate efficiently control *U. decumbens,* while *I. grandifolia* was tolerant to this herbicide, which increased as the soil P content increased.

## Materials and methods

The experiments were conducted in a greenhouse at the Universidade Estadual Paulista, Jaboticabal, Brazil. The experiments with arabica coffee were conducted from October 2018 to July 2019 and those of weeds from May to December 2019. The clay soil used in all experiments, characterized as a Red Latosol, consisted of 59, 20 and 21% clay, silt and sand, respectively. Soil was collected in the 0–20 cm layer in an area with no history of herbicide application. After collection, soil was dried, sieved and a representative sample was taken to analyzes its physical and chemical characteristics (Table 1). Roundup WG (Monsanto, Brazil, ammonium salt, 720 g ae kg^−1^) was the trade formulated used.

**Table 1:** Chemical analysis of the soil, before phosphate fertilization and after 150 days in contact with phosphate fertilizer, Jaboticabal—SP, 2019.

Soil	pH	OM	P	S	Ca	Mg	K	H + Al	SB	CTC	V
	CaCl_2_	g dm^−3^	mg dm^−3^		mmol_c_ dm^−3^						%
**Before phosphate fertilization**											
	6.2	7	6	9	20	6	1	13	27.4	40.3	68
**150 days after phosphate fertilization**											
**Experiment with arabica coffee**											
I	5.5	7	7	18	14	6	0.9	11	20.0	31.2	64
II	6.0	11	15	28	15	5	1.0	14	21.7	35.6	61
III	6.0	11	40	8	20	5	1.0	17	25.2	42.5	59
IV	5.6	8	172	26	26	4	1.0	18	31.8	49.8	64
**Weed experiment**											
V	5.9	6	14	11	31	11	10.8	22	52.1	74.2	70
VI	5.8	7	180	10	34	9	9.4	23	52.4	75.6	69

pH in CaCl_2_ by potentiometry; Organic matter (OM) by Spectrophotometry; P in resin by spectrophotometry; S for turbidimetry; Ca by Atomic Absorption Spectrometry; Mg by Atomic Absorption Spectrometry; K by Atomic Absorption Spectrometry; H + Al in SMP Buffer by potentiometry; Sum of bases (SB) = Ca + Mg + Na + K; cation exchange capacity (CTC) = SB + H + Al; base saturation index (V%) = (SB/CTC) × 100 (Reference: IAC 2001).

### Response of arabica coffee cultivars to glyphosate and soil P content

The coffee cultivars used in the experiments were Catuaí-Vermelho/IAC-144 and Catuaí-Amarelo/IAC-62, which were subjected to three sub-doses of glyphosate [0, 90 and 180 g ha^−1^, equivalent to 0, 5 and 10%, respectively, of the recommended dose for weed control (1800 g ha^−1^)] and four levels of P available in the soil (7, 15, 40 and 172 mg Pi dm^−3^). Experiments were arranged in a factorial scheme (3 glyphosate sub-doses × 4 soil P content), with four repetitions per interaction. Each experimental unit was a polyethylene pot, containing 10.0 dm^3^ of soil and one coffee seedling.

The different P concentrations were achieved by adding 0 (there was already P in the soil), 10, 50 or 100 g of triple superphosphate (46% P and 12% Ca) per pot. The fertilizer was homogenized to the soil prior to transplanting the coffee seedlings. After fertilization, the soil was irrigated daily, in order to maintain 80% of the field capacity. The contact time between the Pi fertilizer and the soil was 150 days (Table 1).

Coffee seedlings (~ 21–22 cm high) were purchased in a commercial nursery. The soil was fertilized as recommended for coffee cultivation^2^, the day of transplanting. Irrigation was carried out daily and maintained as required by the crop. Seedlings, with an average of 16 (Catuaí-Amarelo/IAC-62) and 17 (Catuaí-Vermelho/IAC-144) leaves, were treated with glyphosate 30 days after transplanting and 180 days after Pi fertilization, using a CO_2_ pressurized (3 bar) sprayer, coupled to a quadricycle at 8.0 km h^−1^, equipped with a bar with two spray tips TT 11003 spaced 0.5 m apart, calibrated to deliver 150 L ha^−1^.

### Characterization of the herbicide solution

Herbicide solutions were characterized in terms of pH, electrical conductivity, surface tension of the drop and the mix viscosity. The herbicide solutions were prepared and placed in a 200 mL Becker. Electrical conductivity was measured using a Marte MB-11P bench conductometer (Scientific Mars, Santa Rita do Sapucaí, MG, Brazil). The pH was measured on a bench pH-meter (Quimis Q400RS Bivolt, Diadema, SP, Brazil)^20^. The surface tension was determined using the drop-drop method in a tensiometer (DataPhysics model OCA 15 Plus) equipped with a digital camera with high resolution and temporal definition. The SCA20 software was used for automation and image processing. The surface tension was calculated based on the Yang-Laplace equation^20^. The drops were evaluated second by second for 1 min. For comparison purposes, the time of 10 s was standardized in all treatments to obtain the useful value of the surface tension^20^. Viscosity (mPa s^−1^) was determined on a viscometer (Brookfield, DV-I Prime) at 100 rpm for 20 s. The readings were taken after preparing the herbicide solution.

### Parameters evaluated in coffee plants

Visual intoxication caused by glyphosate in coffee plants, by comparing treated plants with non-treated plant, was assessed at 7, 14, 21, 28 and 35 days after application (DAA). The intoxication scores ranged from 0 to 100%, where 0 (zero) was no intoxication and 100 plant death^38^. In addition, at 7, 14, 21, 28, 35, 42, 63, 77, 84 and 90 DAA, plant height (cm) and stem diameter (mm) were measured. At 35 and 90 DAA, the plants had their organs separated (leaves and stem) to determine the leaf area, dry matter of the aerial part and macronutrients.

Leaf area of each plant was measured by the Li-Cor 3000 m (Li-Cor Instruments, model LI3000A). The parts of the plants were stored in paper bags and dried at 65 ± 2 °C until constant mass. Once weighed, the aerial part was ground in a Wiley-type micro mill (Marconi, TE-840, Brazil) equipped with a sieve (60-mesh), and stored in paper bags to determine the concentration of mineral nutrients (N, P, K, S, Mg and Ca). To determine the N content, the samples were subjected to sulfuric digestion, while to determine the P, S, K, Mg and Ca contents, the samples were subjected to nitro perchloric digestion. After digestion, the levels of N and P were determined by the semi-microkjedahl and colorimetric methods of phosphovanadate-molybdic acid, respectively^39^; the level of K, Mg and Ca was determined by atomic absorption spectrophotometry^40^; and the S content was determined by the turbidimetric method^41^.

### Response of weeds to glyphosate and soil P content

Three seeds of *I. grandifolia* and *U. decumbens*, acquired commercially, were sown per pot (3 dm^3^) and, after emergence, only one plant per pot (experimental unit) was conserved. Thirty g of P_2_O_5_ was added per pot to reach the concentration of 14 mg Pi dm^−3^, and 100 g of P_2_O_5_ for 180 mg Pi dm^−3^. The contact time between the Pi fertilizer and the soil was 150 days. Three doses of glyphosate (0, 1080 and 1800 g ha^−1^) were evaluated, and herbicide was applied on weed plants with 3–4 true leaves, under the conditions previously described for coffee. pH, electrical conductivity, surface tension of the drop and viscosity of the herbicide solutions were also characterized^20^.

Weed response to glyphosate was evaluated in experiments separated per species, in a completely random design with seven repetitions. Visual assessments of percentage control in relation to the control treatment (without herbicide application) were performed, where 0 (zero) corresponds to the absence of intoxication and 100 (one hundred), death of the plant at 28 DAA^38^. The leaf area (only of green leaves), dry matter and P content of the aerial part was determined, as previously described for coffee plants^39–41^.

### Data analysis

Data were analyzed separately for each coffee cultivar and weed species. The characterization of the herbicide solutions had a completely random design with four replications. For height, number of leaves and stem diameter up to 35 DAA, eight repetitions were considered and for the other coffee plant variables, the analyzes were performed with four repetitions.

All data were firstly tested for normality (Shapiro–Wilk test) and homogeneity (Levene's test), and subsequently subjected to ANOVA (F test). Since ANOVA was significant, the means were compared by the post hoc Tukey's test. These tests were performed by using the AgroEstat 1 software (AgroEstat, SP, Brazil) considering a 5% probability of error. When isolated factors were significant, we performed a regression analysis by using the SigmaPlot 10 software (Systat Software, Inc., San Jose, USA, www.systatsoftware.com) basing the choice of models on the significance and the determination coefficient.

### Ethics statement

The authors declare no approvals were required for the study, which complied with all relevant regulations. The development of this research does not include the use of genetically modified and/or threatened plants.

## Supplementary Information

Supplementary Information.

## Data Availability

The datasets generated and/or analyzed during the current study are available from the corresponding author on reasonable request.
