# Supplementary material for: Effect of glyphosate and P on the growth and nutrition of Coffea arabica cultivars and on weed control
Source: Sci Rep. 2021 Apr 14;11:8095. doi: 10.1038/s41598-021-87541-z (PMC8047001; doi:10.1038/s41598-021-87541-z)
Supplement: Supplementary file 1 — Supplementary Information. [file 41598_2021_87541_MOESM1_ESM.docx]

**Effect of glyphosate and P on the growth and nutrition of *Coffea arabica* cultivars and on weed control**

Yanna Karoline Santos da Costa¹*, Nagilla Moraes Ribeiro¹, Guilherme Cesar Pereira de Moura¹, Artur Rodrigues Oliveira¹, Silvano Bianco¹, Ricardo Alcántara-de la Cruz², Leonardo Bianco de Carvalho¹,

^1^São Paulo State University (UNESP), School of Agricultural and Veterinarian Sciences, 14884-900, Jaboticabal, SP, Brazil.

^2^ Chemistry Departament, Federal University of São Carlos, São Carlos 13565-905, São Paulo, Brazil.

*Email: [yanna.costa@unesp.br](mailto:yanna.costa@unesp.br), Phone: +55 16 3209-7100/7916

**Supplementary materials**

**
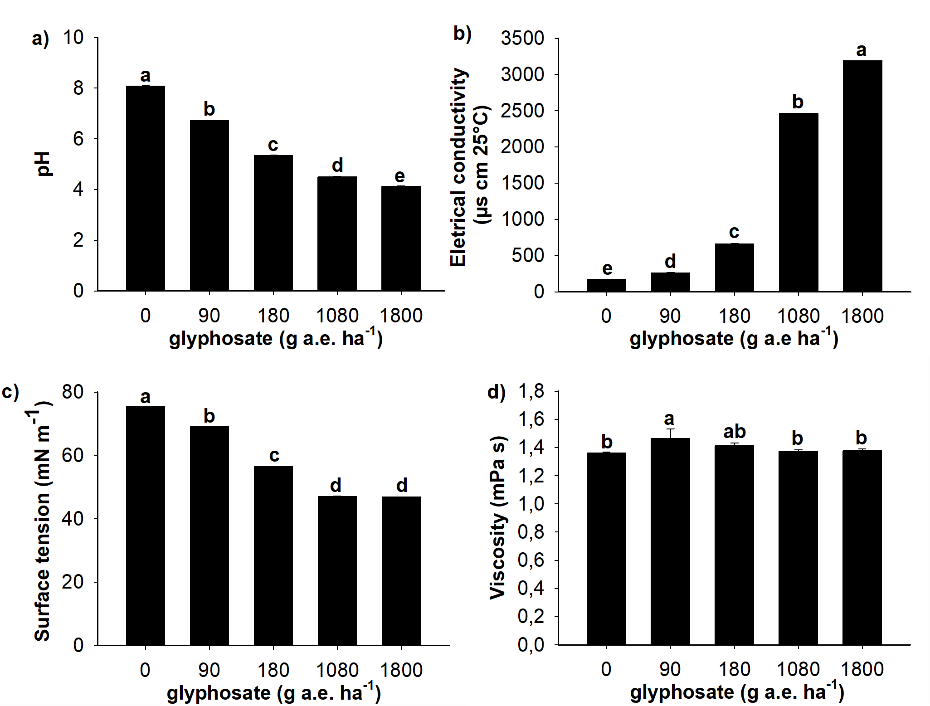
**

**Figure S1.** Characterization of herbicide solution sprayed on coffee and weeds regarding pH (a), electrical conductivity (b), surface tension of the drop (mN m^-1^) (c), viscosity (d) and glyphosate dose (0 (tap water), 90 and 180 g ea ha^-1^). Same lowercase letters do not differ by Tukey's test at 5% probability. Vertical bars indicate the standard deviation (*n*= 4). Plots were drawn using SigmaPlot ver. 10.0 (Systat Software, Inc., San Jose, USA, www.systatsoftware.com).

**Figure S2**. Number of leaves in the Arabica coffee cultivars Catuaí-Amarelo/IAC-62 (a, c) and Catuaí-Vermelho/IAC-144 (b,d) cultivated in soils with different content of P (mg dm^-3^) and subjected to subdoses of glyphosate in relation to the days after glyphosate application. For each evaluation period, means followed by * were different by Tukey's test at 5% probability. Plots were drawn using SigmaPlot ver. 10.0 (Systat Software, Inc., San Jose, USA, www.systatsoftware.com).

**Figure S3.** Average nutrient content of the shoot (g kg^-1^ plant^-1^) of the arabica coffee cultivars Catuaí-Amarelo/IAC-62 and Catuaí-Vermelho/IAC-144, cultivated in soils with different levels of P and subjected to subdoses of glyphosate at 35 days after application. a and g) Nitrogen; b and h) sulfur; c and i) calcium; d and j) phosphorus; e and k) potassium; and f and l) magnesium. For each nutrient, same lower-case do not differ between the soil P contents, and same upper-cases do not differ between the glyphosate subdoses by the Tukey test at 5% probability. ± Vertical bars indicate the standard deviation (*n*= 4). Plots were drawn using SigmaPlot ver. 10.0 (Systat Software, Inc., San Jose, USA, www.systatsoftware.com).

**Figure S4.** Leaf area (cm^2^ plant^-1^) of *Ipomoea grandifolia* and *Urochloa decumbens* plants grown in soils with different content of P and without glyphosate application. Plots were drawn using SigmaPlot ver. 10.0 (Systat Software, Inc., San Jose, USA, www.systatsoftware.com).
